# A soil property and spectral near- and mid-infrared dataset from cultivated fields sampled in winter 2020, Western Highveld, South Africa

**DOI:** 10.1016/j.dib.2026.113160

**Published:** 2026-08-10

**Authors:** Anru-Louis Kock, George Van Zijl

**Affiliations:** Unit for Environmental Sciences and Management, North-West University, Potchefstroom Campus, Building E6, 11 Hoffman Street, North West Province, Potchefstroom, 2531, South Africa

**Keywords:** Spectroscopy, Machine learning, Combined spectra, Open data

## Abstract

This data article describes a soil dataset of 790 topsoil samples collected from commercial, cultivated grain-producing fields managed by NWK Ltd. And GWK Ltd. in the Western Highveld region, South Africa. Samples were composite topsoil drawn from the co-operative’s routine fertility advisory submissions during a single 2020 winter sampling season; they therefore represent the soil fertility ranges of actively cultivated fields rather than natural profiles. The dataset is provided in a single *.csv* file and contains reference data for soil pH (KCl), Phosphorus (P-Bray 1), and exchangeable cations Potassium (K), Calcium (Ca) Magnesium (Mg) and Sodium (Na). For each sample, the dataset includes two corresponding spectra: (1) handheld Near-Infrared (NIR) spectra (approx. 7 407 – 4 003 cm⁻¹) acquired with a NeoSpectra spectrometer, and Mid-Infrared (MIR) spectra (approx. 3997 – 600 cm⁻¹) acquired with a Bruker Alpha II spectrometer. This data is valuable for developing and validating pedometric models, for NIR and MIR instrument comparison and spectral fusion, and for extending soil spectral libraries in the Southern African region. The data are openly available in the Zenodo repository within the “Pedometrics South Africa” community.

Specifications Table

**Table:** 

Subject	Soil Science, Agronomy and Crop Science, Analytical Chemistry (Spectroscopy)
Specific subject area	Pedometrics – soil fertility characterisation and diffuse-reflectance (NIR and MIR) soil spectroscopy of cultivated soils.
Type of data	Table (single .csv file). Raw, analysed (converted) and merged tabular data. Figure (Locality map, spectral plot).
How data was acquired	Topsoil samples were collected by NWK and GWK during routine fertility sampling. Reference chemistry was determined by an ISO/IEC ISO/IEC 17,025:2017 accredited laboratory (NviroTek Laboratories): pH in 1:2.5 soil:1 M KCl; plant-available P by the Bray-1 method; exchangeable K, Ca, Mg and Na by 1 M ammonium acetate (NH₄OAc, pH 7) extraction [1]. MIR spectra: Bruker Alpha II spectrometer with a DRIFT module, 2 cm⁻¹ resolution, 3 997 – 600 cm⁻¹ [1,2]. NIR spectra: NeoSpectra handheld FT-NIR spectrometer (Si-Ware Systems), 1 350 – 2 500 nm reflectance [3] Full protocols are in “Experimental design, materials and methods”.
Data format	Raw; Analysed and Merged.
Data source location	Cultivated grain fields in the Western Highveld, North West Province, South Africa (NWK Ltd. service area). Field coordinates (WGS84) spanning **24.83°–26.62° E, 25.91°–26.66° S** (Lichtenburg–Coligny–Delareyville–Ottosdal district; see Fig. 1), coordinates were not released for the remaining samples.
Data accessibility	The data is deposited in the Zenodo.org repository within the “Pedometrics South Africa” community. Repository name: Zenodo Data identification number: zenodo.17,510,653**.** Direct URL to data: https://doi.org/10.5281/zenodo.17510653
Related research article	Kock, A.-L., Ramphisa-Nghondzweni, P. D., & Van Zijl, G. (2024). Development of soil spectroscopy models for the Western Highveld region, South Africa: Why do we need local data? *European Journal of Soil Science*, 75(6), e70014. https://doi.org/10.1111/ejss.70014

## 1 Value of the Data

• The dataset provides a paired NIR and MIR comparison on identical soil samples, enabling direct benchmarking of a low-cost handheld NIR sensor (NeoSpectra) against a higher-precision benchtop MIR instrument (Bruker Alpha II) for the same commercial reference methods.
• Both spectra are reported on a common absorbance / wavenumber (cm^–1^) axis with a documented conversion, the data can be used with or without further pre-processing to develop and validate spectral fusion and transfer-learning models for key soil fertility parameters.
• The data fills a documented regional gap. The Western Highveld grain belt and the South African pH (KCl) reference method are under-represented in existing global spectral libraries and extend the public “Pedometrics South Africa” library.
• The MIR spectra and reference chemistry underpin a peer-reviewed study [1] giving users an external validation context and a reproducible link between the raw data and published model performance.
• The complete acquisition and processing workflow (instruments, resolutions, unit and reflectance-to-absorbance conversions, merging code) is documented here so that other laboratories can reproduce the dataset structure and append comparable samples.

## 2 Background

Diffuse-reflectance soil spectroscopy in the near-infrared (NIR) and mid-infrared (MIR) regions is an established, rapid and low-cost alternative to conventional wet chemistry for characterising soil properties, and its performance across a wide range of physical, chemical and biological attributes has been reviewed extensively [[4], [5], [6]]. The predictive value of spectroscopy depends heavily on large, representative calibration libraries: global efforts such as the Africa Soil Information Service (AfSIS) MIR library and the Open Soil Spectral Library (OSSL) have demonstrated both the promise of shared spectral data and the strong dependence of model accuracy on local representation [[7], [8], [9], [10]]. Where a region or a specific reference method is poorly represented, global models systematically mis-predict local samples and flag them as outside the calibration feature space [1,10].

South African contributions to these global libraries remain limited, and cultivated soils of the Western Highveld grain belt are essentially absent from them [1]. In addition, the pH (KCl) determination routinely used in South African commercial soil analysis is not represented in the global model collections, which provide only pH (H₂O) and pH (CaCl₂) calibrations [1]. Our earlier work showed that locally calibrated MIR models for this region outperform corresponding global models for pH, Ca and Mg, underscoring the value of regional reference data [1], a pattern consistent with international findings that local or “spiked” calibrations improve prediction over purely global models [9,11]. The comparatively recent availability of low-cost handheld NIR sensors further motivates paired NIR–MIR datasets, because such instruments could lower the barrier to routine on-site soil assessment if their performance relative to benchtop MIR is quantified [6,12].

Against this background, the present dataset was compiled to (i) make the underlying soil property and MIR spectral measurements of Kock et al. [1] openly available, and (ii) extend them with paired handheld NIR spectra collected for the same samples. The combined dataset enables direct comparison between low-cost handheld NIR and high-precision benchtop MIR measurements, supports the development of NIR and MIR spectral-fusion models, and establishes a growing, openly licensed South African soil spectral library.

## 3 Data Description

The dataset is provided as a single comma-separated value file, *NWU_NIR_MIR_Dataset.csv*. Each of the 790 rows is one soil sample; columns are as follows (Table 1, Table 2).

**Table 1:** Identifier and reference-chemistry columns.

Column	Description	Unit
Sample Name	Unique sample identifier (e.g., G23–46,926).	–
pH (KCl)	Soil pH in 1:2.5 soil:1 M KCl.	pH units
P (Bray 1)	Plant-available phosphorus (Bray-1).	mg·kg⁻¹
K	Exchangeable potassium (1 M NH₄OAc).	mg·kg⁻¹
Ca	Exchangeable calcium (1 M NH₄OAc).	mg·kg⁻¹
Mg	Exchangeable magnesium (1 M NH₄OAc).	mg·kg⁻¹
Na	Exchangeable sodium (1 M NH₄OAc).	mg·kg⁻¹

**Table 2:** Summary of statistics of the soil properties.

Soil property	Mean (μ)	Median	Σ	Min	Max	Range
pH (KCl)	5.25	5.19	0.80	3.69	7.62	3.93
Bray-1 P (mg·kg⁻¹)	26.26	19.00	24.12	0.82	255.68	254.85
Exchangeable K (mg·kg⁻¹)	148.34	134.00	76.82	2.00	526.32	524.32
Exchangeable Ca (mg·kg⁻¹)	472.24	424.50	255.93	45.00	2243.03	2198.03
Exchangeable Mg (mg·kg⁻¹)	117.99	102.44	70.56	16.00	461.63	445.63
Exchangeable Na (mg·kg⁻¹)	11.72	9.00	7.86	0.52	84.58	84.06

σ = Standard deviation; Min = Minimum; Max = Maximum.

### 3.1 Spectral columns

The spectral columns are named spec.<wavenumber> and run from spec.7407 to spec.600 in cm⁻¹. All spectral values are reported as absorbance. Two instruments contribute contiguous, non-overlapping wavenumber blocks on this common axis:

• NIR block – spec.7407 to spec.4003 (≈7 407– 4 003 cm⁻¹; equivalently 1 350 – 2 500 nm), from the NeoSpectra handheld FT-NIR; reflectance percentage.
• MIR block – spec.3997 to spec.600, from the Bruker Alpha II (DRIFT), reported at 2 cm⁻¹ spacing.

## 4 Experimental Design, Materials and Methods

### 4.1 Sample collection and preparation

**Fig. 1:**
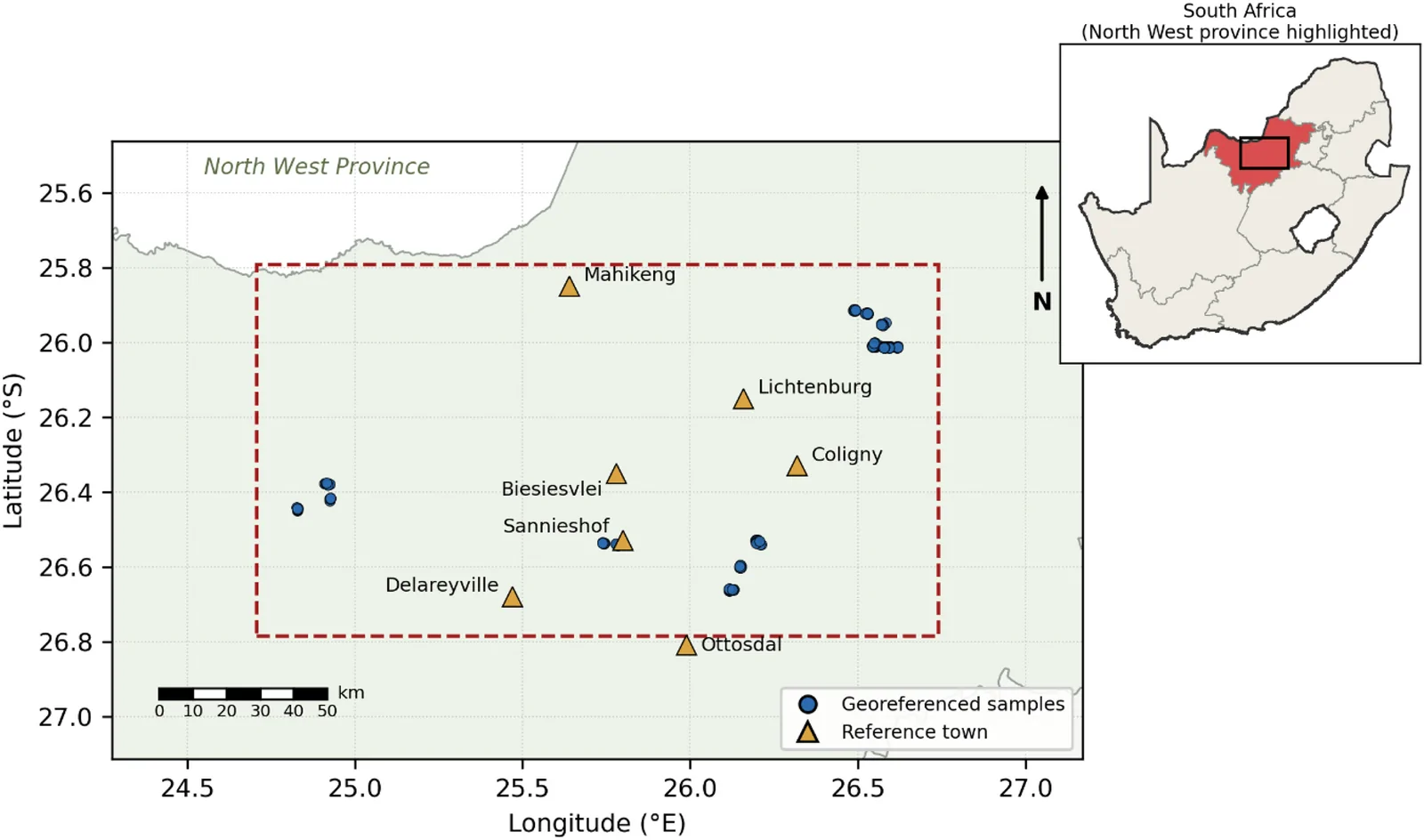
Locality of the soil samples in the Western Highveld, North West Province, South Africa. Blue circles are the 178 samples for which field coordinates (WGS84) were released by the data provider (many coincide at the field scale), orange triangles are reference towns for orientation, the dashed rectangle is the coordinate extent of the georeferenced samples (≈24.83–26.62° E, 25.91–26.66° S). Field coordinates were not released for the samples.

Soil samples were obtained from North West Kooperasie (NWK) and Griekwaland Wes Kooperasie (GWK), two agricultural service co-operatives that carry out routine fertility sampling for commercial grain producers in the Western Highveld. The 790 samples used here were a subset of the co-operative’s seasonal fertility advisory submissions for a single 2020 winter sampling season [1]. Each field topsoil sample was taken across the mapped management unit to a depth of 0–15 cm using auger/spade, following the co-operative’s standard sampling protocol. Field coordinates (WGS84) were released by the provider for 178 samples and are mapped in Fig. 1. For the remaining samples the commercial providers did not release georeferenced field locations, so only co-operative and regional attribution is available (see Limitations).

### 4.2 Laboratory (reference) analysis

Soil chemical properties were determined by NviroTek Laboratories using standard procedures:

– pH (KCl): 1:2.5 soil:1 M KCl suspension.
– P (Bray-1): Bray-1 extraction; P determined by colorimetry.
– Exchangeable K, Ca, Mg, Na: 1 M ammonium acetate (NH₄OAc, pH 7) extraction.
– All nutrient concentrations (P, K, Ca, Mg, Na) reported in mg·kg⁻¹.

### 4.3 Spectral acquisition

#### 4.3.1 Mid-infrared (MIR) spectroscopy

MIR spectra were collected as described by Kock et al. [1] using a Bruker Alpha II spectrometer with a DRIFT (diffuse reflectance) module [2]. Samples were oven-dried (60 °C, 24 h), ground with a motorised mortar and pestle and passed through a 100 µm sieve. Spectra were collected at 2 cm⁻¹ resolution over 3997 – 600 cm⁻¹ (reported in the CSV as spec.3 997 – spec.600) as the co-added average of 30 scans. Background measurements were taken regularly with a gold reference cap to compensate for atmospheric and instrumental drift.

#### 3.3.2 Near-infrared (NIR) spectroscopy

The dataset was extended with NIR spectra for the same samples. Samples were oven-dried before scanning. NIR reflectance was measured with a handheld NeoSpectra FT-NIR spectrometer (Si-Ware Systems) [3]: the 100 µm -sieved soil was placed in a sample holder and the scanner placed directly on the sample surface. A single 15 s scan was acquired per sample via the NeoSpectra Collect Android application, recording reflectance (R) over 1350–2500 nm A white reference standard was scanned before each sample batch to calibrate the device and correct for sensor drift.

#### 3.3.3 Data processing and merging

Raw NIR reflectance (1 350 – 2 500 nm) was harmonised with the MIR data by wavelength to wavenumber conversion, cm⁻¹ = 10⁷ / nm, giving ≈7 407 – 4 003 cm⁻¹. The processed NIR block was then merged with the MIR spectra and reference values by unique sample ID using the base *merge()* function in R [13].

### 3.4 Technical validation

**Fig. 2:**
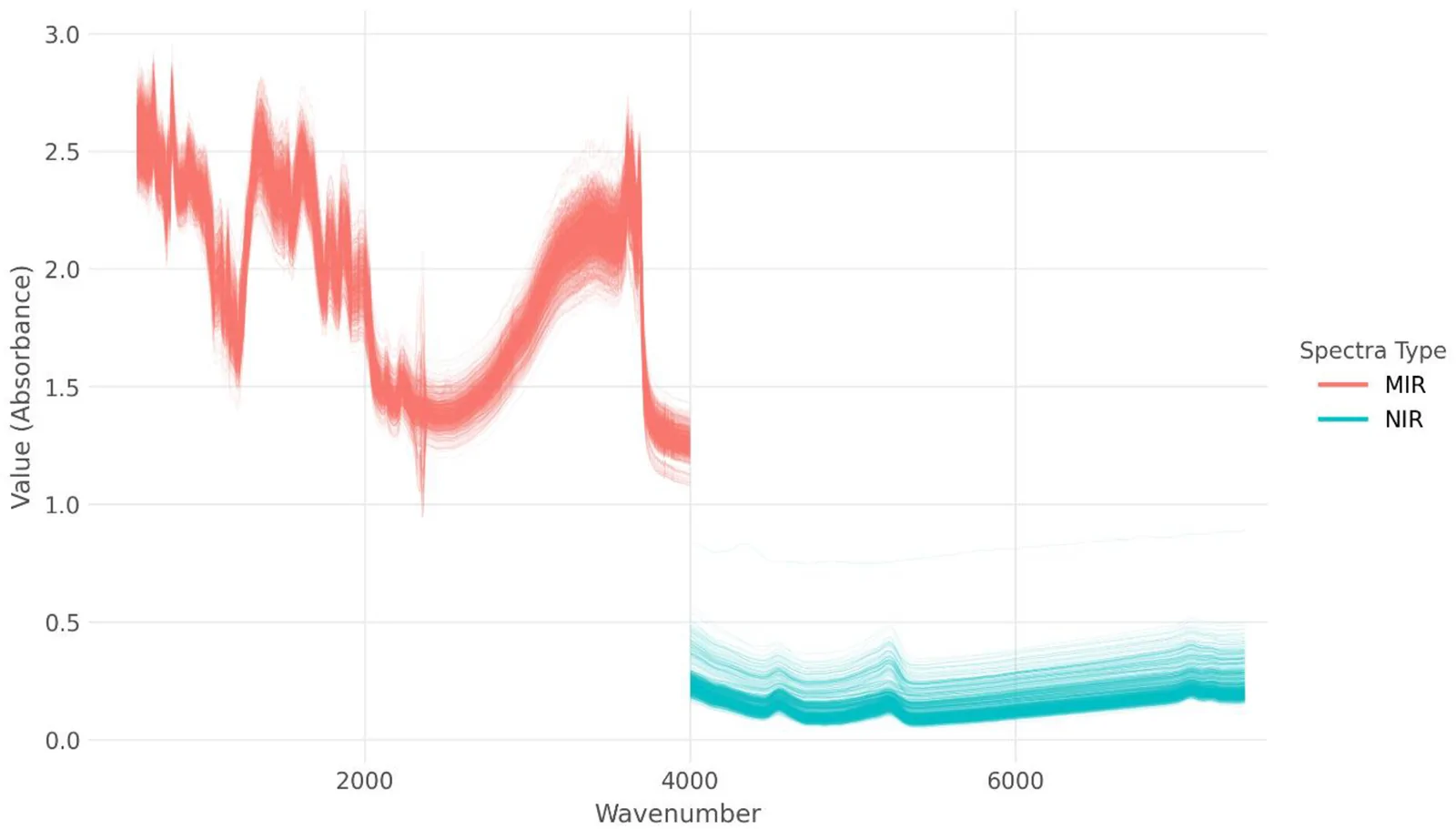
Spectral plot of all samples that show the merged spectral data of the absorbance values of the MIR spectra and the converted absorbance values of the NIR spectral data for this illustration.

The reliability of the dataset was ensured through a multi-step validation process. The chemical reference data was generated by an accredited laboratory (NviroTek) adhering to standard operating procedures. For the spectral data, a visual inspection of the combined NIR and MIR plots was conducted to identify and remove any samples exhibiting spectral artifacts, saturation, or significant noise. Thirteen samples with flat/blank MIR spectra were excluded, giving n = 790. Fig. 2 illustrates the continuity and quality of the merged spectra for five random samples, demonstrating the alignment between the converted NIR absorbance data and the MIR absorbance data.

## Limitations

The dataset is geographically restricted to the Western Highveld region of North West Province and was collected from cultivated croplands managed by two service providers (NWK Ltd. and GWK Ltd.). The samples represent the soil property ranges typical of commercial grain producing fields in this semi-arid region and are not representative of natural soils or other land uses in South Africa. Field coordinates (WGS84) were available for a subset of 178 samples (mapped in Fig. 1); for the remaining samples the commercial service providers, under their client confidentiality terms, released only the broader regional extent rather than georeferenced field locations. Coordinates are therefore incomplete and not included in the public dataset, and users requiring fully georeferenced data should note this partial coverage.

## Ethics Statement

The authors have read and followed the ethical requirements for publication in Data in Brief and confirming that the current work does not involve human subjects, animal experiments, or any data collected from social media platforms.

## Ethics Declaration

The author(s) declare(s) that the study does not involve humans nor animal subjects.

## Credit Author Statement

Anru-Louis Kock: Investigation, Visualization, Methodology, Data curation, Software, Validation, Writing, Original draft preparation. George Van Zijl: Conceptualization, Supervision, Reviewing and Editing.

## Data Availability

ZenodoWestern Highveld Soil fertility, NIR and MIR spectral data (Original data).
